# Effect of Phase Changes on the Axial Modulus of an FeMnSi-Shape Memory Alloy

**DOI:** 10.3390/ma14174815

**Published:** 2021-08-26

**Authors:** Yajiao Yang, Matteo Breveglieri, Moslem Shahverdi

**Affiliations:** 1 Empa, Swiss Federal Laboratories for Materials Science and Technology, 8600 Dübendorf, Switzerland; yajiao.yang@empa.ch (Y.Y.); matteo.breveglieri@empa.ch (M.B.); 2 School of Civil Engineering, University of Tehran, Tehran 4563-11155, Iran

**Keywords:** FeMnSi-based shape memory alloys, axial modulus *E_SMA_*(*κ*), activation process, phase transformation, plastic deformation

## Abstract

The axial modulus *E_SMA_*(*κ*) of FeMnSi-based shape memory alloys (FeMnSi-SMAs) is a parameter introduced in this study to characterize the relationship between stress and strain behavior at the early stage of tensile loading. *E_SMA_*(*κ*) can be used to correctly estimate and model the interaction forces between FeMnSi-SMAs and other materials. Unlike the conventional Young’s modulus, which is usually given at room temperature, the *E_SMA_*(*κ*) is evaluated at different temperatures and strongly depends on phase transformation and plastic deformation. This study investigated the evolution of *E_SMA_*(*κ*) during and after pre-straining as well as in the course of the activation processes. The effect of different factors (e.g., phase transformation and plastic deformation) on the magnitude of *E_SMA_*(*κ*) is discussed. The result shows that the *E_SMA_*(*κ*) can differ significantly during activation and thus needs to be modified when interaction forces between FeMnSi-SMAs and other substrates materials (e.g., concrete) must be modeled and evaluated.

## 1. Introduction

Shape memory alloys (SMAs) are unique alloys that can recover their shapes caused by deformation through unloading or upon heating above a specific temperature. Because of the shape memory effect (SME) and superelasticity, SMAs are widely applied in the automotive, aerospace, robotic, civil engineering, and biomedical domains [1,2,3,4,5,6,7,8,9]. NiTi-based SMAs (NiTi-SMAs) have played a leading role in the industry owing to their good SME. However, they are high-priced, implying that they cannot be easily employed in large amounts as required in civil engineering. As an alternative to NiTi-SMAs, the FeMnSi-based SMA (FeMnSi-SMA) has attracted considerable attention over the past two decades due to its low cost, good machinability, workability, and weldability [10]. For structural applications, a big advantage of an iron-based SMA is the significantly higher Young’s modulus in comparison to NiTi-based products [11].

The mechanism governing the SME of a FeMnSi-SMA is the phase transformation between γ-austenite (face-centered cubic, FCC) and ɛ-martensite (hexagonal close-packed, HCP), which is induced by the application of external stress and/or temperature changes. The undeformed FeMnSi-SMA mainly contains an unstable γ-austenite phase at room temperature. The γ-austenite phase can transform into ɛ-martensite with the application of external stress, and finally, change the shape of FeMnSi-SMA. The stress-induced ɛ-martensite can go back to γ-austenite upon heating above the austenite starting temperature *A_S_*, and consequently, FeMnSi-SMA recovers its shape to some extent. Such a characteristic of FeMnSi-SMA has attracted significant attention from the industry.

**Figure 1:**
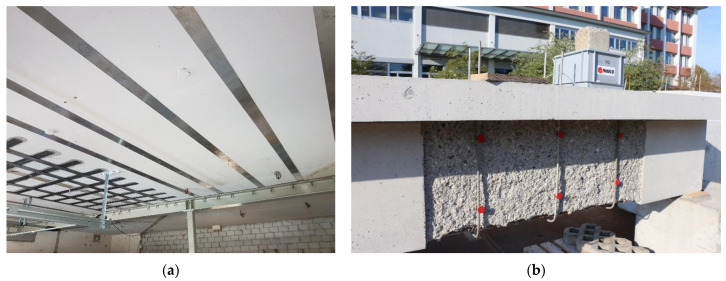
Application of FeMnSi-SMAs in civil engineering: (**a**) an example of FeMnSi-SMA strips used as a flexural strengthening of a reinforced concrete slab. (**b**) FeMnSi-SMA bars applied to improve the shear carrying capacity of a RC beam.

In civil engineering, FeMnSi-SMAs have been used to prestress reinforced concrete members, such as slabs and bridge girders [12]. The prestressing effect, which develops after the SMA heating, keeps the concrete members in compression or in case of flexure and reduces the tensile strains, thus preventing or reducing crack propagation. It decreases the deflection of the structural elements and increases their service life [13,14,15,16,17]. Figure 1a shows, as an example, FeMnSi-SMA strips used as a flexural strengthening of a reinforced concrete slab, while Figure 1b depicts FeMnSi-SMAs’ bars applied to improve the shear carrying capacity of a beam. In the first application, the strips are constrained at the extremities; meanwhile, in the second, Fe-SMAs will be embedded in a cementitious shotcrete layer. For conventional prestressing elements, e.g., steels or carbon fiber reinforced polymers (CFRPs), complex anchoring and hydraulic systems are needed to induce prestress force [12]. However, in the case of FeMnSi-SMAs, the prestressing phase is simplified as there is no need to use sophisticated mechanical systems due to the shape memory effect.

**Figure 2:**
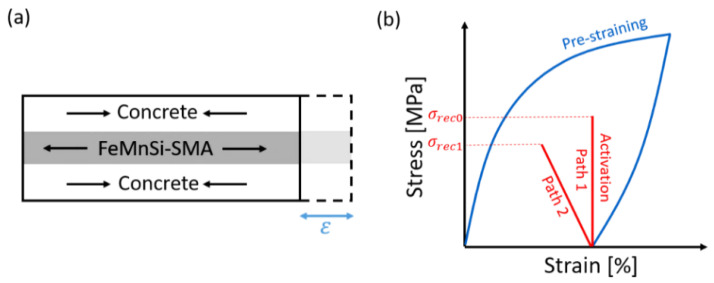
Schematic illustration of the interaction force between the concrete and the FeMnSi-SMA strip. (**a**) Example of activated FeMnSi-SMA material embedded into concrete, and (**b**) typical FeMnSi-SMA stress-strain diagram during the pre-straining and activation phase.

The prestressing effect in FeMnSi-SMAs is achieved by following three main steps. Step 1: pre-straining, which consists of deforming the FeMnSi-SMA material to the desired strain level to generate stress-induced martensite, followed by unloading after the deformation. Step 2: similar to a conventional steelwork, the FeMnSi-SMA strip can be fixed at its ends to the concrete [18], as shown in Figure 1a or alternatively, in the case of bars, it can be embedded in an additional cementitious layer (or shotcrete) [19], as shown in Figure 2a. Step 3: the SMA is heated up to a target temperature and cooled down to room temperature. This thermal process is called activation. 

Since the FeMnSi-SMA cannot return to its original configuration, stress is developed due to the reverse martensitic phase transformation upon heating (as illustrated in Figure 2a). The total stress achieved in the FeMnSi-SMA after the activation and cooling to room temperature is called recovery stress, *σ_rec_*.

When FeMnSi-SMA strips or bars are fixed at their ends, the evolution of recovery stress during the activation process is shown as Path 1 in Figure 2b. However, in the case of non-fully rigid constraint, for example, flexible parent structure in Figure 1a [20], the evolution of the recovery stress obeys Path 2. In concrete-embedded FeMnSi-SMA, due to the concrete contraction, the recovery stress can be, for this reason, slightly lower. The relative stiffness between SMA and substrate rules the iteration forces between the materials. Their interaction can be expressed as a function of α [21]:

(1) $$\alpha =\frac{k_{C}}{k_{SMA}}=\frac{k_{C}L}{A_{SMA}}\cdot \frac{1}{E_{SMA}\left(\kappa \right)}$$

where, *k_C_* and *k_SMA_* are the axial stiffness of the concrete and the FeMnSi-SMA element, respectively; *A_SMA_* is the SMA cross-section area, and *L* is the reference length. During activation, the values of *A_SMA_*, *k_C_*, and *L* can be considered constant. *E_SMA_*(*κ*), however, introduced in the present study as axial modulus, is a variable that depends on the path of the thermo-mechanical process. *κ* in *E_SMA_*(*κ*) indicates a different state during pre-straining and activation. For example, *E_SMA_*(*κ*_0_) is the *E_SMA_* before pre-straining; *E_SMA_*(*κ*_1_) is the *E_SMA_* of the pre-strained specimen; *E_SMA_* (50 °C *↑*) is the *E_SMA_* at 50 °C in the heating process during activation. It is essential to investigate how the *E_SMA_*(*κ*) develops to correctly estimate and model the interaction forces between FeMnSi-SMAs and the other materials. Among numerous studies about FeMnSi-SMAs, there is no research dedicated to the study of the *E_SMA_*(*κ*), except some preliminary investigations reported by the present authors in a recent study [21]. This study aims to more comprehensively investigate the *E_SMA_*(*κ*) of the novel FeMnSi-SMA.

## 2. Materials and Methods

The investigated material in this study has the composition of Fe-17Mn-5Si-10Cr-4Ni-1(V, C) wt%. It is a hot rolled sheet material (re-fer, Seewen, Switzerland)) with a final cold rolling treatment to reduce the sheet thickness to 1.5 mm. The specimens consist of FeMnSi-SMA strips, which were laser-cut into a final geometry equal to 250 mm of length, a width of 15 mm, and a thickness of 1.5 mm. The experiment includes two parts. The axial modulus *E_SMA_*(*κ*) was evaluated before and after pre-straining (first part), and during the activation process (second part).

### 2.1. Test Procedure for Determining E_SMA_(κ_0_) before Pre-Straining and E_SMA_(κ_1_) after Pre-Straining

**Figure 3:**
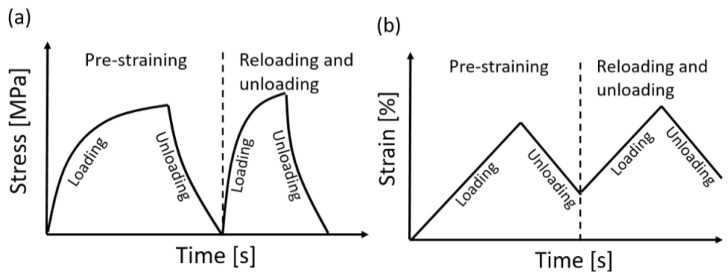
Schematic illustration of the evolution of (**a**) stress and (**b**) strain as a function of time during pre-straining and re-loading experiments for determining *E_SMA_*(*κ*_0_) and *E_SMA_*(*κ*_1_).

**Figure 4:**
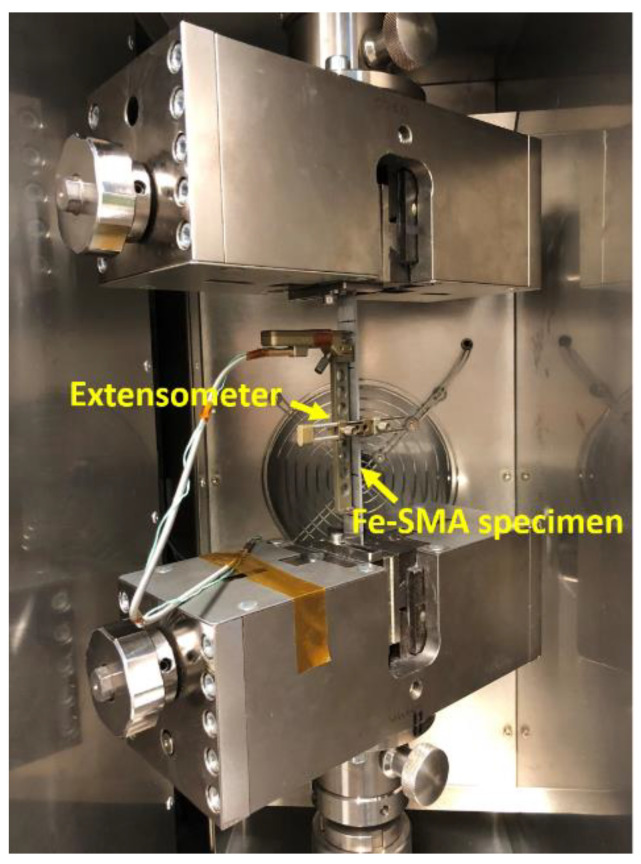
Inner part of the climate chamber on a Z020 Zwick machine: a FeMnSi-SMA specimen was fixed between two clamps, and the clip-on Mini MFA 2 extensometer was installed on the specimen.

The axial modulus of the as-received specimen *E_SMA_*(*κ*_0_) was calculated from the stress-strain curve of the tensile loading experiment. The pre-straining tests consist of loading the specimen to 2% or 4% and progressively unloading it with the strain rate of 1%/min. The explanatory stress versus time curve and strain versus time curve during pre-straining can be seen in Figure 3a,b, respectively. The strain values of 2% and 4% were chosen based on previous investigation [22]. The pre-straining experiments were conducted on a 250 kN Zwick machine at room temperature. After the pre-straining test, the specimen was re-loaded to 1% to determine the axial modulus of the pre-strained specimen *E_SMA_*(*κ*_1_) (Figure 3). Re-loading and unloading were carried out on a Z020 machine (Zwick, Germany) equipped with a climate-controlled chamber (Figure 4). The FeMnSi-SMA specimen was fixed between two clamps, and a clip-on Mini MFA 2 extensometer equipped with an extension arm was installed on the specimen. The gauge length was 100 mm. Both tests were performed at room temperatures of approximately 23 °C.

### 2.2. Test Procedure for the Determination of E_SMA_(κ) during Activation

**Figure 5:**
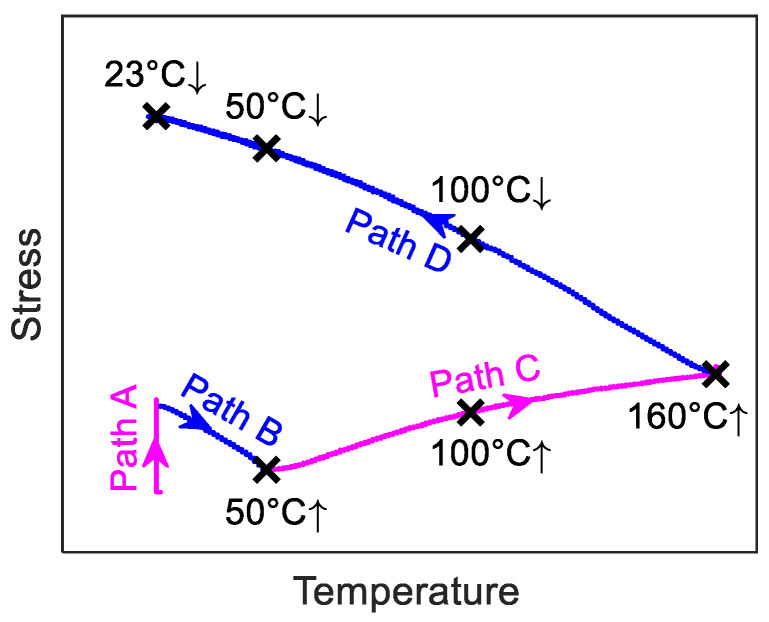
Stress versus temperature curve during the activation process. The FeMnSi-SMA is heated up from 23 °C to 160 °C and then cooled down to 23 °C. The black cross-marks show the target temperatures of the interrupted thermal-mechanical experiments.

*E_SMA_*(*κ*) assessment during activation was carried out using the Z020 Zwick machine, equipped with a climate-controlled chamber and the Mini MFA 2 extensometers (Figure 4). All the specimens were previously pre-strained to 2 or 4%. Figure 5 shows an example of a stress-temperature curve during the full activation process. At the beginning of activation (path A), the FeMnSi-SMA specimen was pre-loaded to 50 MPa, followed an additional displacement of 10 µm to avoid compressive stresses during heating because of the impeded thermal expansion. During the heating process, the axial stress first decreases with the increase of temperature (path B), because the FeMnSi-SMA expands. As the temperature increases, the stress is recovered (path C). This effect is caused by the combined effect of ɛ→γ phase transformation and thermal expansion. The maximum temperature 160 °C is commonly chosen since at this temperature, significant recovery stress (about 300 MPa) can be achieved without causing any damage to the concrete [22]. During the cooling process (path D), the axial stress increases with decreasing temperature, which involves thermal contraction of the specimen, phase transformation, and plastic deformation.

**Figure 6:**
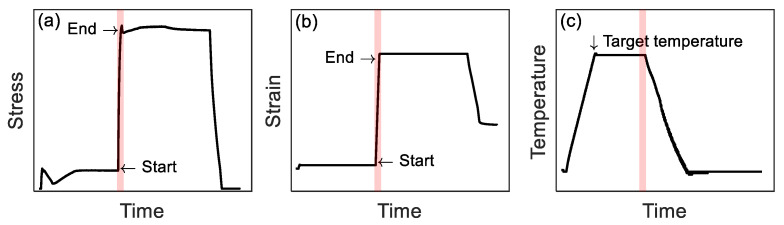
Protocol of the interrupted activation experiment to determine the *E_SMA_*(*κ*). (**a**) Stress, (**b**) strain, and (**c**) temperature versus experimental time curves, respectively.

The *E_SMA_*(*κ*) during activation was determined by interrupting the activation process and by performing a tensile test at the selected activation step. Figure 6 shows the stress (a), strain (b), and temperature (c) versus time in a thermo-mechanical experiment interrupted at a target temperature, respectively. Under the strain-control conditions, specimens were heated up from 23 °C to a target temperature at a thermal rate of 2 °C/min (Figure 6c). After one hour of waiting at the constant target temperature, specimens were loaded to 1% strain at a 0.2 mm/min rate to determine the *E_SMA_*(*κ*). The loading process is marked by “Start” and “End” in Figure 6a,b (red shaded area in Figure 6). Finally, specimens were cooled down and unloaded. Table 1 lists the target temperatures (test temperatures): 50 °C ↑, 100 °C ↑, and 160 °C ↑ during heating, and 100 °C ↓, 50 °C ↓ and 23 °C ↓ during cooling (‘↑’ and ‘↓’ indicate that the thermal cycle was interrupted during heating ‘↑’ or cooling ‘↓’). The target temperatures are also depicted in Figure 5. The selection of 50 °C ↑ was because it approximately corresponds to the minimum stress value during the activation process. A temperature between 50 °C ↑ and 160 °C ↑ was selected, i.e., 100 °C ↑. Accordingly, 100 °C ↓ and 50 °C ↓ were chosen during the cooling process.

### 2.3. Mechanisms Governing the Value of E_SMA_(κ)

During the tensile loading of a normal austenitic stainless steel (without SME), the material will first undergo elastic deformation, which means that the specimen can recover its dimension upon removing the load. In the elastic deformation regime, the external stress leads to the movement of atoms from their equilibrium position but retains their relative positions to each other. Once the stress is removed, all atoms move back to their equilibrium positions. Young’s modulus can be derived from the slope of the regression line that fits the experimental data in the elastic loading range of the stress-strain curve. The yield stress represents the upper limit of the elastic regime. It is expected that when the stress reaches this yield stress, plastic deformation involving dislocation movement takes place, and the shape of the specimen cannot be recovered upon removing the load.

For the FeMnSi-SMA, the stress-induced ɛ-martensite can form with the application of stress at the very beginning of loading [23]. The martensite nucleus can be considered a single stacking fault with a supplementary displacement, which results in a volume dilatation of the FeMnSi-SMA because of martensitic phase transformation [24]. Therefore, besides the movement of atoms from their equilibrium position at the beginning of loading, the supplementary displacement caused by martensite formation will additionally contribute to the measured strain. As a result, at the same stress level, a larger strain is measured for the FeMnSi-SMA compared to a normal austenitic stainless steel. Consequently, a smaller slope of the regression line fitting at the very beginning of the stress-strain curve can be observed, resulting in a smaller modulus value. More γ→ɛ phase transformation can contribute more to the measured strain, and lead to a lower modulus value. As a result, for the FeMnSi-SMA, the measured slope cannot be simply considered as Young’s modulus since the γ→ɛ phase transformation is involved. Therefore, axial modulus *E_SMA_*(*κ*) is used in this study to characterize the relationship between stress and strain at the early stage of loading instead of Young’s modulus.

In general, the *E_SMA_*(*κ*) depends on γ→ɛ phase transformation, plastic deformation, initial microstructure (the fraction of austenite and martensite), and temperature. Plastic deformation affects the *E_SMA_*(*κ*) by dislocation movement. When the applied stress reaches the yield stress, dislocations start to move, which contributes to the measured strain and affects the *E_SMA_*(*κ*). It should be noted that plastic deformation only plays an important role in determining the *E_SMA_*(*κ*) when the axial stress gets close to the yield stress of the material at the beginning of loading. For example, for the measurement of *E_SMA_*(*κ*) during the cooling process of activation, the axial stress is very high and even reaches the yield stress, and therefore the plastic deformation can already occur at the start of tensile loading (the details can be found in Section 3.2). The temperature and initial microstructure (fraction of austenite and martensite) influence the *E_SMA_*(*κ*) mainly by affecting the Young’s modulus of the material. In general, the Young’s modulus of steel decreases with temperature [25]. On the other hand, the fraction of martensite and austenite phases in the material can affect material’s Young’s modulus due to the difference of Young’s modulus for these two phases. However, such an effect does not play an essential role in determining the axial modulus because of the following reasons and therefore is neglected. Firstly, during pre-straining, only small amount of stress-induced ɛ-martensite is generated, e.g., about 10% [26]. Furthermore, the difference of Young’s modulus is small for austenite and martensite. For example, the Young’s modulus of γ-austenite *E_A_* estimated from type 316 austenitic steel is about 207 GPa [27]. The Young’s modulus of ɛ-martensite *E_M_* estimated from a type 10% Cr martensite steel is about 214 GPa [28]. Therefore, the effect of different amount of ɛ-martensite and γ-austenite on material’s modulus can be neglected in this study. In a short summary, the *E_SMA_*(*κ*) is mainly determined by phase transformation, plastic deformation and temperature.

## 3. Results and Discussion

### 3.1. The E_SMA_(κ) before and after Pre-Straining

**Figure 7:**
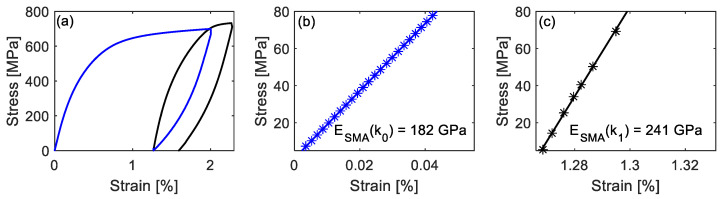
(**a**) Stress as a function of strain during pre-straining (blue) and re-loading (black); (**b**) the linear fitting (regression line) in 5–80 MPa of pre-straining; (**c**) the linear fitting (regression line) in 5–80 MPa of re-loading.

Figure 7a shows the stress-strain curve to determine specimen’s axial modulus before and after pre-straining. The blue line shows loading to 2% and unloading to 0 MPa during pre-straining. In the unloading process, the stress-strain curve diverges from a straight line. The diverged strain is the pseudoelasticity *ɛ_pe_*, which is due to back transformation from ɛ-martensite to γ-austenite and a reversible motion of Schockley partial dislocations upon unloading [23,29,30,31]. After pre-straining, the specimen was re-loaded to 1% and unloaded (black line) to evaluate *E_SMA_*(*κ*_1_).

The axial modulus is determined by calculating the slope of linear regression, which most closely fits the initial data of the stress-strain experimental curve. For the sake of consistency, a stress range between 5 and 80 MPa is chosen for all the axial modulus evaluations in this study. The blue line and stars in Figure 7b show data points in the 5–80 MPa stress range during pre-straining (blue line in Figure 7a) and the regression line, respectively. The slope of this regression line is the axial modulus of the as-received specimen *E_SMA_*(*κ*_0_). *E_SMA_*(*κ*_0_) is determined to be 180 GPa (standard deviation is 6 GPa) by averaging the values from 19 experiments. The detailed results of these experiments can be found in Table 2. *R*^2^ shows the goodness-of-fit for the linear regression models and are all higher than 0.999. Similarly, Figure 7c presents data points in the stress range of 5–80 MPa of the re-loading after pre-straining, and the black line is its regression line with slope *E_SMA_*(*κ*_1_) equal to 241 GPa, which is larger than *E_SMA_*(*κ*_0_).

For the as-received material, during pre-straining, some unstable γ-austenite transforms into ɛ-martensite owing to the external stress application. After pre-straining, the amount of unstable γ-austenite is less in the pre-strained specimen compared to the as-received one. During re-loading, because of the loss of unstable γ-austenite, it is expected that less γ→ɛ phase transformation takes place in the pre-strained specimen, resulting in a steeper slope at the beginning of the stress-strain curve, and accordingly, a larger *E_SMA_*(*κ*_1_) than *E_SMA_*(*κ*_0_).

On the other hand, it can be seen that the stress during re-loading is higher than that during the initial pre-straining. This is because more ɛ-martensite was generated after pre-straining, resulting in more significant hardening during the subsequent re-loading.

### 3.2. The E_SMA_ (50 °C ↑) and E_SMA_ (50 °C ↓) in Activation

**Figure 8:**
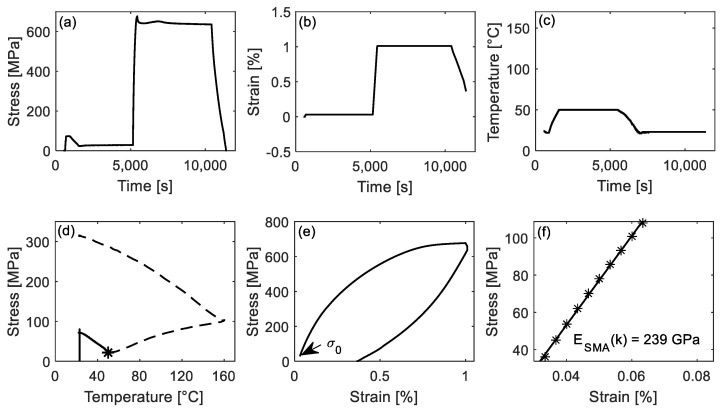
(**a**–**c**) Stress, strain and temperature versus time curves of the interrupted experiments to determine *E_SMA_* (50 °C *↑*). (**d**) Stress as a function of temperature during activation. The black star (50 °C ↑) marks the point, at which *E_SMA_* was evaluated. (**e**) Stress-strain curve during loading to 1% strain and unloading. (**f**) The linear fitting (regression line) in the stress range from *σ*_0_ + 5 to *σ*_0_ + 80 MPa.

Figure 8 shows the results for the determination of *E_SMA_*(*κ*) at 50 °C ↑. Figure 8a–c depict the stress, strain, and temperature versus time during the *E_SMA_*(*κ*) evaluation test. The solid line in Figure 8d presents the stress-temperature curve, and the dashed line depicts the plausible development of a full activation process. The black star marks the testing point (50 °C ↑), at which *E_SMA_* (50 °C ↑) is evaluated. Figure 8e demonstrates the stress-strain curve during loading to 1% strain. The initial axial stress *σ*_0_ at the beginning of loading, pointed by the arrow, is 29 MPa (also corresponds to the black star in Figure 8d). The *E_SMA_* (50 °C ↑) is determined by fitting a regression line in the stress ranging from *σ*_0_ + 5 MPa to *σ*_0_ + 80 MPa (the same stress range was used in Section 3.1, which corresponds to a stress range of 34 to 109 MPa. Figure 8f shows the data points used for the fitting of the regression line. The calculated *E_SMA_* (50 °C *↑*) is 239 GPa.

**Figure 9:**
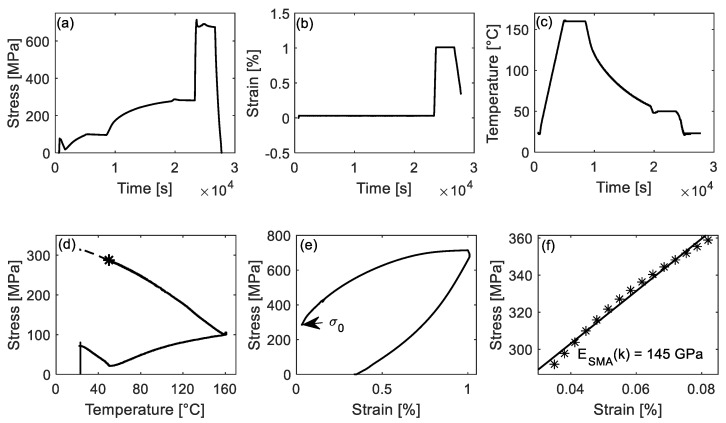
(**a**–**c**) Stress, strain, and temperature versus time curves of the interrupted experiments to determine *E_SMA_*(*50* *°C ↓*). (**d**) Stress as a function of temperature during activation. The black star (50 °C ↓) marks the point, at which *E_SMA_* was evaluated. (**e**) Stress-strain curve during loading to 1% strain and unloading. (**f**) The linear fitting (regression line) in the stress range from *σ*_0_ + 5 to *σ*_0_ + 80 MPa.

Similarly, the assessment of the *E_SMA_* (50 °C *↓*) can be seen in Figure 9a–f. The *σ*_0_ at 50 °C ↓ is 281 MPa, and therefore the stress range for the regression is 286–361 MPa. The resulting *E_SMA_* (50 °C *↓*) is 145 GPa.

During the activation process from 50 °C ↑ to 50 °C ↓, the thermal expansion and contraction effect of the FeMnSi-SMA on the axial stress can be canceled out due to the same temperature value. Since the results show a larger *σ*_0_ at 50 °C ↓ than that for 50 °C ↑, it is expected that the ɛ→γ phase transformation is dominant during activation and there is more austenite in the specimen at 50 °C ↓ than that for 50 °C ↑. Consequently, the γ→ ɛ phase transformation is more likely to happen at 50 °C ↓ and a lower *E_SMA_*(*κ*) is observed as mentioned in Section 3.1. In addition, the higher *σ*_0_ at 50 °C ↓ can enhance the martensitic phase transformation during loading to 1% and further lower *E_SMA_*(*κ*). On the other hand and at 50 °C ↓, the stress range chosen for fitting the regression line is 286–361 MPa. The yield stress of the original specimen is 224 MPa. This means the regression line is fitted in the stress range of plastic deformation. The measured strain at this stage may involve phase transformation and dislocation movement, which further contributes to the measured strain and leads to a lower slope.

### 3.3. The E_SMA_(κ) during the Activation

**Figure 10:**
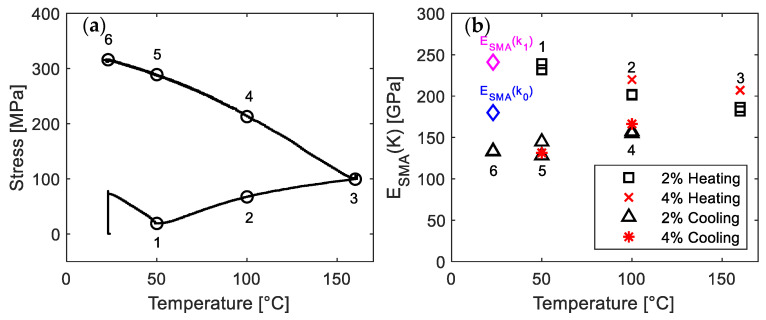
Evolution of *E_SMA_*(*κ*) in activation. (**a**) Number 1–6 show the interrupted temperatures during activation. (**b**) The corresponding *E_SMA_*(*κ*).

Figure 10 summarizes the results of *E_SMA_*(*κ*) during activation. Figure 10a shows the points marked by numbers 1–6, at which the activation process was interrupted, and *E_SMA_* was evaluated. Figure 10b depicts the corresponding *E_SMA_*(*κ*). The black squares and triangles represent the *E_SMA_*(*κ*) with 2% pre-straining during the heating and cooling process, respectively. It can be seen that the *E_SMA_*(*κ*) decreases during the heating process and keeps decreasing within the cooling process until the thermal cycle is complete (23 °C ↓). The *E_SMA_*(*κ*_0_) and *E_SMA_*(*κ*_1_) are also depicted for comparison. As discussed above, *E_SMA_*(*κ*_1_) is larger than *E_SMA_*(*κ*_0_). All the experimental values of *E_SMA_*(*κ*) together with *σ*_0_ and *R*^2^ can be found in Table 3.

**Figure 11:**
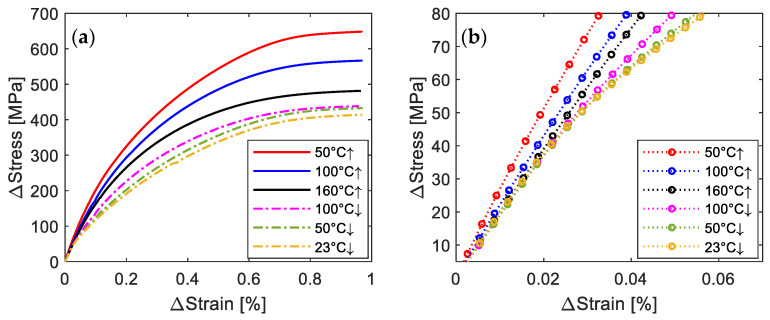
(**a**) Representative Δstress-Δstrain curves during loading to 1%. The initial Δstress/Δstrain is set to be zero. (**b**) The experimental data in the stress range of 5–80 MPa.

Figure 11a comprises the Δ*σ* vs. Δ*ε* (Δ*σ* = *σ* − *σ*_0_; Δ*ε* = *ε* − *ε*_0_) at the beginning of loading phases to determine the *E_SMA_*(*κ*). All the presented specimens were pre-strained to 2%. Since the *σ*_0_ is affected by the temperature path, the values are expressed in terms of increment. Figure 11b shows the data points in the stress range of 5–80 MPa. It is shown that the slope of the curves (i.e., *E_SMA_*(*κ*)) is decreasing as the entire activation proceeds. It can also be seen in Figure 11b that significant plastic deformation occurs for the states during the cooling of activation process (i.e., 23 °C ↓, 50 °C ↓ and 100 °C ↓), and hence leads to lower *E_SMA_*(*κ*).

During the heating process, both *σ*_0_ and the fraction of austenite keep increasing after the point 50 °C ↑. Consequently, the higher *σ*_0_ and larger amount of austenite for the later stage of the heating process can result in a larger extent of the phase transformation during the tensile loading, and hence lead to smaller *E_SMA_*(*κ*). On the other hand, during the cooling, the values of *σ*_0_ may reach the yield stress of the material, and therefore plastic deformation can occur at the early stage of the tensile experiment, which is expected to contribute to the reduction of *E_SMA_*(*κ*) with decreasing temperature. It should be noted that the Young’s modulus increases with decreasing temperature for the normal material without SME. The observed smaller *E_SMA_*(*κ*) at lower temperature during the cooling of activation process indicates that the effect of phase transformation and plastic deformation on *E_SMA_*(*κ*) is more significant than that of temperature for the presented case.

**Figure 12:**
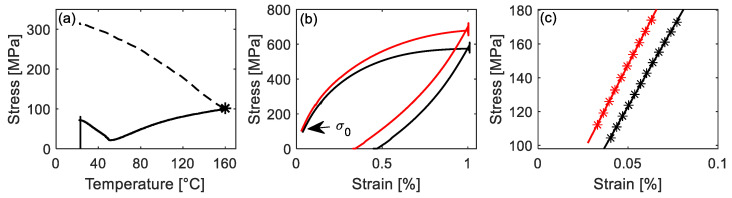
(**a**) Stress vs. temperature up to the target temperature (The dashed line shows the expected path for full activation). The black star (50 °C ↓) marks the point, at which *E_SMA_* was evaluated. (**b**) Stress-strain curve to determine the *E_SMA_*(*κ*). (**c**) The linear fitting (regression line) in the stress range from *σ*_0_ + 5 to *σ*_0_ + 80 MPa. Red and black lines in (**b**,**c**) represent the specimen pre-strained to 4% and 2%, respectively.

The *E_SMA_*(*κ*) of the specimens pre-strained to 4% was indicated by red cross-marks (heating process) and red star-marks (cooling process) in Figure 10b. Similar to the trend observed for the specimens pre-strained to 2%, the *E_SMA_*(*κ*) keeps decreasing until the thermal cycle is complete. However, compared to 2% pre-strained specimens, the values of *E_SMA_*(*κ*) for the specimens pre-strained to 4% are slightly larger at the same heating temperature. For example, Figure 12a–c compare the determination of *E_SMA_*(*κ*) at 160 °C ↑ for the specimens pre-strained to 2% (black color) and 4% (red color). It can be seen in Figure 12b that the initial axial stresses *σ*_0_ of both specimens are almost equal, but the specimen pre-strained to 4% shows a higher slope (red line in Figure 12b,c). This is because pre-straining to 4% can result in a larger extent of phase transformation from unstable austenite to martensite in comparison to 2% pre-straining, which makes the γ→ɛ phase transformation during the loading to 1% at the *E_SMA_* testing point more difficult and results in a higher *E_SMA_*(*κ*).

## 4. Conclusions and Outlook

In this study, the axial modulus *E_SMA_*(*κ*) of FeMnSi-SMAs has been investigated during and after pre-straining as well as in the course of the activation processes. The experimental observations can be summarized as follows:

- The *E_SMA_*(*κ*) is mainly determined by phase transformation, plastic deformation and temperature.
- The axial modulus during the re-loading after pre-straining is larger than that during the pre-straining. This is because the amount of unstable γ-austenite is less in the pre-strained specimen compared to the as-received alloy, resulting in less γ→ɛ phase transformation in the pre-strained specimen at the beginning of the stress-strain curve, and accordingly, a larger *E_SMA_*(*κ*_1_) than *E_SMA_*(*κ*_0_).
- The evolution of *E_SMA_*(*κ*) during the activation process of the specimens initially pre-strained to 2% and 4% keeps decreasing as the activation process proceeds until the thermal cycle is done. At the later stage of the activation process and during the tensile loading for *E_SMA_*(*κ*) evaluation, more γ→ɛ phase transformation and plastic deformation can occur, and therefore lead to a smaller *E_SMA_*(*κ*).
- The values of *E_SMA_*(*κ*) for the specimens initially pre-strained to 4% are slightly larger than those for the specimens initially pre-strained to 2% at the same heating temperature. This is because pre-straining to 4% can result in a larger extent of γ→ɛ phase transformation (and plastic deformation) in comparison to 2% pre-straining, and therefore lead to less γ→ɛ phase transformation during the tensile loading at the *E_SMA_* testing point, i.e., a higher *E_SMA_*(*κ*).
- Microstructure investigation (e.g., electron backscattered diffraction and X-ray diffraction) is recommended in future work to characterize the phase transformation during the pre-straining and activation process.

## Figures and Tables

**Table 1:** List of pre-straining and interrupted thermomechanical experiments. The target temperatures of interrupted thermomechanical experiments are 50 °C ↑, 100 °C ↑, and 160 °C ↑ during heating, and 100 °C ↓, 50 °C ↓, and 23 °C ↓ during cooling (‘↑’ and ‘↓’ indicate that the thermal cycle was interrupted during heating ‘↑’ or cooling ‘↓’).

Process	Interrupted Temperature [°C]	Symbol	Pre-Straining [%]	Experiment Number
Heating	50	50 °C ↑	2	2
	100	100 °C ↑	2	2
			4	1
	160	160 °C ↑	2	2
			4	1
Cooling	100	100 °C ↓	2	2
			4	1
	50	50 °C ↓	2	2
			4	1
	23	23 °C ↓	2	2

**Table 2:** The detailed *E_SMA_*(*κ*_0_) values of 19 pre-straining experiments.

Pre-Straining to 2%						Pre-Straining to 4%	
*E_SMA_*(*κ*_0_) [GPa]	$R^{2}$	*E_SMA_*(*κ*_0_) [GPa]	$R^{2}$	*E_SMA_*(*κ*_0_) [GPa]	$R^{2}$	*E_SMA_*(*κ*_0_) [GPa]	$R^{2}$
175	0.9998	170	0.9999	184	1.0000	173	0.9999
174	0.9999	188	1.0000	182	1.0000	183	0.9998
182	1.0000	184	0.9999	171	0.9999	188	0.9999
189	0.9998	184	1.0000	182	1.0000	185	1.0000
178	0.9999	173	1.0000			178	1.0000

**Table 3:** The values of *σ*_0_, *E_SMA_*(*κ*), and *R*^2^ at all experimental temperatures in activation.

Pre-Straining [%]	Temperature [°C]	*σ*_0_ [MPa]	*E_SMA_*κ [GPa]	*R* ^2^
2	50 °C ↑	29	239	0.999
2		24	232	0.998
2	100 °C ↑	68	202	1.000 ^A^
2		71	201	0.999
4		75	220	0.998
2	160 °C ↑	92	182	1.000 ^A^
2		93	186	0.999
4		100	207	0.999
2	100 °C ↓	217	157	0.995
2		219	155	0.985
4		225	166	0.994
2	50 °C ↓	286	128	0.962
2		281	145	0.997
4		287	131	0.958
2	23 °C ↓	312	133	0.983
2		311	134	0.983

^A^ Only two experimental points were used for the calculation of the regression.

## Data Availability

All raw/processed data necessary for reproducing results in this study can be accessed on reasonable request.
